# Characterization of microglial transcriptomes in the brain and spinal cord of mice in early and late experimental autoimmune encephalomyelitis using a RiboTag strategy

**DOI:** 10.1038/s41598-021-93590-1

**Published:** 2021-07-12

**Authors:** Shaona Acharjee, Paul M. K. Gordon, Benjamin H. Lee, Justin Read, Matthew L. Workentine, Keith A. Sharkey, Quentin J. Pittman

**Affiliations:** 1 Hotchkiss Brain Institute, Snyder Institute for Chronic Diseases, Department of Physiology and Pharmacology, Cumming School of Medicine, University of Calgary, 3330 Hospital Drive NW, Calgary, AB T2N 4N1 Canada; 2 Centre for Health Genomics and Informatics, Cumming School of Medicine, University of Calgary, Calgary, AB Canada; 3 Department of Comparative Biology and Experimental Medicine, Faculty of Veterinary Medicine, University of Calgary, Calgary, AB Canada

**Keywords:** Glial biology, Neuroimmunology, Synaptic transmission, Multiple sclerosis

## Abstract

Microglia play an important role in the pathogenesis of multiple sclerosis and the mouse model of MS, experimental autoimmune encephalomyelitis (EAE). To more fully understand the role of microglia in EAE we characterized microglial transcriptomes before the onset of motor symptoms (pre-onset) and during symptomatic EAE. We compared the transcriptome in brain, where behavioral changes are initiated, and spinal cord, where damage is revealed as motor and sensory deficits. We used a RiboTag strategy to characterize ribosome-bound mRNA only in microglia without incurring possible transcriptional changes after cell isolation. Brain and spinal cord samples clustered separately at both stages of EAE, indicating regional heterogeneity. Differences in gene expression were observed in the brain and spinal cord of pre-onset and symptomatic animals with most profound effects in the spinal cord of symptomatic animals. Canonical pathway analysis revealed changes in neuroinflammatory pathways, immune functions and enhanced cell division in both pre-onset and symptomatic brain and spinal cord. We also observed a continuum of many pathways at pre-onset stage that continue into the symptomatic stage of EAE. Our results provide additional evidence of regional and temporal heterogeneity in microglial gene expression patterns that may help in understanding mechanisms underlying various symptomology in MS.

## Introduction

Microglia are the resident myeloid cells in the CNS that play an important role in brain function in both homeostatic and disease states^1^. In healthy and developing brain, microglia interact with neurons and play a significant role in synaptic pruning, neuronal wiring and synaptic plasticity^2^. These cells are also the “first responders” to changes in homeostasis in the brain parenchyma^3^. Under physiological conditions, microglia exist in a ramified state and are highly motile as they extend their fine processes to survey their surroundings. When microglia become activated in response to changes in homeostasis, they retract their processes and become more ameboid^4^. However, this morphological correlate of microglial activation state has been challenged in the past decade^5^. Transcriptional profiling of microglia has contributed immensely to the understanding of the microglial function^6^. Microglial activation has been associated with several neurological disorders including Alzheimer’s disease^7^, amyotrophic lateral sclerosis^8^ and multiple sclerosis^9^.

Multiple sclerosis (MS) is an inflammatory disease of the nervous system characterized by cerebral and spinal cord white matter demyelination, inflammation and axon injury and degeneration^10^. MS may either be associated with, or occur independently of neuronal loss^11, 12^ that results in sensory and motor impairments. While MS may exist as more than one subtype^13, 14^, experimental autoimmune encephalomyelitis (EAE) is an animal model of MS that recapitulates several features of MS including motor and sensory alterations associated with demyelination, synaptic and neuronal loss and inflammation of the CNS^15–18^. EAE is induced by provoking an antibody response using a myelin associated antigen given in conjunction with Complete Freund’s adjuvant and pertussis toxin. The latter two compounds provide an early innate immune response to initiate a subsequent T cell invasion and attack of CNS myelin. We and others found that EAE animals develop changes in coping and anxiety-like behaviour prior to the onset of motor symptoms (the pre-onset stage)^19–24^. As we found changes in synaptic function at this early stage of EAE, we explored the contribution of microglia because of their known involvement in synaptic pruning^25^. We found that early, pre-symptomatic EAE was associated with an altered microglia phenotype in the basolateral nucleus of the amygdala^26^. At the electron microscopic level, we found increased process area, increased perimeter and reduced extracellular digestion in the microglia of EAE animals compared to controls, which was indicative of a de-activated phenotype^26, 27^. This observation contrasted with the expected, activated microglia phenotype usually found in inflammation^1^. Even though these animals showed behavioural changes, we did not observe any T cell invasion and demyelination in the brain at this early stage of disease^19^. In contrast, later stages of the disease, (associated with profound motor impairment) feature the well-known T cell invasion, synaptic impairment and associated demyelination^18^. This continuum of innate (in early EAE) and acquired (late EAE) immune response provides an opportunity to characterize microglial phenotype under different inflammatory conditions. This is made relevant by the appreciation of significant interactions between the innate and acquired immune responses within the brain in MS^28^. Furthermore, the progression of the inflammatory immune response in EAE in different parts of the central nervous system allows us to expand on the increasing evidence for phenotypic and functional differences in microglial populations by comparing brain and spinal cord. This is important because disease pathology in the grey matter of central nervous system is quite different from that of the largely white matter of the spinal cord^11, 12^ and there is also compelling evidence that microglia derived from the cortex and spinal cord display different phenotypes^29^.

In order to better understand the microglial activation state during pre-onset and symptomatic stages of a disease, we have conducted a transcriptomic profile of the microglia in the brain and spinal cord from EAE animals using RiboTag technology. This technology allowed us to isolate ribosome-bound mRNA from the frozen brains by immunoprecipitation, without tissue dissociation methods, which has been shown to induce microglial activation markers and also has the potential to be associated with cargo contaminations and transcripts that are sequestered from the ribosomes^30^. Furthermore, it has been suggested that the traditional mechanical dissociation and isolation methodology may not capture the ribosomes that are located in the fine processes of the microglia^31^. Thus, isolating mRNA bound to ribosomes enabled us to study only the RNA that is ready to be translated rather than the global transcriptome of microglia. Using RiboTag technology, we compared microglial transcriptomic profiles of the brain and spinal cord in both the pre-onset stage of EAE, when behavioral alterations, but not motor difficulties are observed, and in symptomatic animals that display gross motor disability associated with demyelination. While there has been a previous study on microglial transcriptomics in EAE (although not using RiboTag approaches)^32^—it used pooled spinal cord and CNS tissues, thus not differentiating between microglial functions associated with the motor and sensory changes involving the spinal cord, and those associated with the behavioral changes that would be more evident in higher brain regions. To the best of our knowledge, this is the first study comparing the transcriptomic profile of microglia in the pre-onset and symptomatic brain and spinal cord in EAE.

## Methods

### EAE induction in mice

All animal protocols were approved by the Health Sciences Animal Care Committee of the University of Calgary, following the guidelines of the Canadian Council on Animal Care. All experiments and use of animals complied with the ARRIVE guidelines. Female mice were used throughout the study as the incidence of MS in females versus males is 3:1^10^. Eight-ten week-old C57BL/6 N female mice (Charles River Laboratories, Montreal, QC, Canada), housed under specific pathogen free conditions, 4/cage, on a 12:12 h light cycle were used in this study. EAE was induced by subcutaneous immunization with 100 μg myelin oligodendrocyte glycoprotein (MOG_35–55_) in emulsion of a 1:1 volume with complete Freund’s adjuvant (CFA, containing 4 mg/ml of heat killed *Mycobacterium tuberculosis*, Difco Laboratories, Detroit, MI, USA). The mice were also injected with intravenous *Bordetella pertussis* toxin (200 ng; PTX, List Biological Laboratories Inc., Campbell, California, USA) in 1X Phosphate Buffered Saline (PBS) at the time of, and two days following, immunization^33^. In each cohort of mice, subjects were randomly chosen for either the EAE or naive group so that equal numbers of each group were represented. Animals were assessed for motor deficits associated with EAE as previously described^19^; animals described as “pre-onset” that were used 8 days after EAE induction were verified to lack the motor weakness that typically emerges only after 10–13 days in our hands. Symptomatic animals had a score of 2–3 (on a 5 point scale) as previously described^19^ and showed tail and hindlimb paralysis. For all experiments, we used three naïve animals, three pre-onset animals and three symptomatic animals.

### Generation of *Cx3cr1*^*CreER*^*: Rpl22*^*HA*^ mice and tamoxifen injection

**Figure 1:**
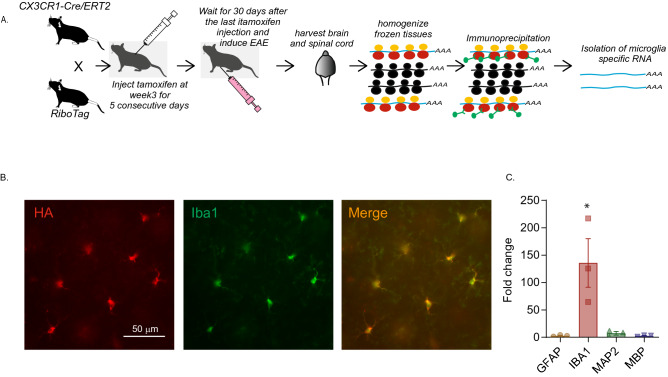
Isolation and validation of microglia-specific mRNA from Cx3cr1^CreER^: Rpl22^HA^ mice. (**A**) Overview of the experimental strategy of generation of *Cx3cr1*^*CreER*^*: Rpl22*^*HA*^ mice and isolating RNA. Black ribosomes in the cartoon represent endogenous wt ribosomes, whereas yellow/red are HA tagged transgenic Rpl22 containing ribosomes. (**B**) Representative images from the amygdala of *Cx3cr1*^*CreER*^*: Rpl22*^*HA*^ mice validating the specificity of HA expression in microglia. CX3CR1 + cells carry GFP and an antibody against GFP was used to label CX3CR1 + cells (green) and anti-HA was used to label Rpl22^HA^ expressing cells. The merged imaged shows that HA and GFP are co-localized. (**C**) Bar graph shows that RNA is significantly enriched in the immunoprecipitated RNA. *indicates *p* < 0.05 (Tukey’s multiple comparison test). The enrichment in the microglial IP RNA was compared to the total RNA before IP. Three animals per group were used in the experiments described in (**B**,**C**).

Female homozygote *Rlp22*^*HA*^ mice (B6N.129-*Rpl22*^*tm1.1Psam*^/J, Stock # 011,029, Jackson Laboratories, Ellsworth, Maine, USA)^34^ were crossed with male homozygote *Cx3cr1*^*CreER*^(B6.129P2(Cg)-*Cx3cr1*^*tm2.1(cre/ERT2)Litt*^*/*WganJ, Stock # 021,160, Jackson Laboratories) mice to generate *Cx3cr1*^*CreER*^*: Rpl22*^*HA*^ mice^30^. The floxed *Rlp22*^*HA*^ allele bearing mice carry three copies of hemagglutinin (HA) epitope that is inserted after a ribosomal protein *Rlp22* allele with a floxed C-terminal, and the HA terminal is positioned just before the stop codon^34^. CX3CR1, a chemokine receptor, is highly expressed in myeloid cells including monocytes and macrophages and has been widely used as a microglial marker^35, 36^. In the *Cx3cr1*^*CreER*^ allele bearing mice, Cre recombinase is expressed under the control of the *Cx3cr1* promoter, and tamoxifen injection allows the estrogen receptor fused Cre-recombinase to gain entry into the nucleus and cause loxP dependent recombination. Mice homozygous for *Rlp22*^*HA*^ were crossed with *Cx3cr1*^*CreER*^ mice to generate *Rlp22*^*HA*^:*Cx3cr1*^*CreER*^ mice, which were then treated with tamoxifen to induce *Rlp22*^*HA*^ expression in the microglia/macrophages (Fig. 1A). In active EAE lesions, a substantial proportion of myeloid cells represent invading macrophages, rather than resident microglia^37^ and the relative proportions may change throughout the disease state^32^. Thus, it is important to be able to differentiate between these two populations. To achieve this, and to isolate the RNA from the microglia only, we took advantage of the fact that microglia have slower turnover rates compared to monocytes and macrophages^38^. Thus, at 3 weeks of age, the offspring received intraperitoneal injection of 1 mg of tamoxifen, dissolved in corn oil, for 5 consecutive days. We then induced EAE in females 30 days after the last tamoxifen injection to restrict the Cre mediated recombination to microglia^39^. In the 30-day interval following the tamoxifen exposure, the monocytes and macrophages expressing HA would have turned over^39^, while HA expressing microglia persisted (Fig. 1A).

### Microglial RNA isolation protocol

The *Cx3cr1*^*CreER*^*: Rpl22*^*HA*^ mice were anesthetized with sodium pentobarbital (80 mg/kg, i.p.) and the lumbar spinal cord and subcortical regions of the brain anterior to the pons (including amygdala, hippocampus, thalamus, basal ganglia and hypothalamus, but not the cerebral cortex, cerebellum or brainstem) were microdissected on ice and snap-frozen in dry-ice. RNA was isolated from the fresh frozen samples using the TRAP (translating ribosome affinity purification) approach^40^ based on a published protocol^34^ with some modifications. Briefly, the tissue was homogenized in polysome buffer and the supernatant from the lysate was incubated with 50 µl of anti-HA magnetic beads (Miltenyi, Auburn, CA, USA) for 2 h at 4 ˚C. Bead bound RNA was recovered by magnet, washed with high salt buffer three times and purified using RNeasy Micro Kit (Qiagen, Valencia, CA, USA).

### RNA enrichment assay

In order to verify that the RNA isolated by the RiboTag method is enriched for the transcripts from microglia, we compared the relative amount of mRNA of a microglial marker gene (*Iba1*) as well as non-microglial marker genes (*Gfap* for astrocytes, *Map2* for neurons and *Mbp* for oligodendrocytes) between the RiboTag RNA (immunoprecipitated (IP) RNA) and the RNA from the whole tissue (Input RNA) by qPCR. An equal amount of RNA from the IP RNA and input RNA was used to generate cDNA using the High Capacity cDNA Reverse Transcription Kit (Applied Biosystems, Burlington, ON, Canada). qPCR was performed using TaqMan Gene Expression assay kit (Applied Biosystems) with the probes for the following cell type specific marker genes; *Iba1* (Mm00479862), *Gfap* (Mm01253033), *Map2* (Mm00485231), *Mbp* (Mm01266402) and *Gapdh* (Mm03303349), which was used as an internal standard. Triplicates of each cDNA were amplified by Step One Real Time PCR system (Applied Biosystems). The results were analyzed using Step One Software (Applied Biosystems) and the enrichment for each marker gene was represented as relative quantification (RQ) value reflecting the relative amount of the transcript in the IP RNA compared to input RNA by ΔΔCT method.

### Immunofluorescence

Naive and EAE animals were anesthetized with sodium pentobarbital (80 mg.kg, i.p.), then perfused with PBS and 4% paraformaldehyde (PFA). Whole brains were dissected, and fixed in 4% PFA overnight at 4 °C. The brains were placed in 20% sucrose + PBS solution overnight, embedded in optimal cutting temperature (OCT) compound, and then flash frozen. Blocks were cut into 20 µm sections on a cryostat. The sections were then incubated with anti-GFP (1:250, Abcam, Toronto, ON, Cat.#5450; validated by the supplier using fluorescent immunocytochemistry) and goat anti-HA (1:250, Abcam, Cat. # ab9110; validated by the supplier by fluorescent immunohistochemistry) overnight. The tissues were washed in PBS and incubated with a donkey anti-rabbit-Cy3 antibody (1:200, Jackson ImmunoResearch Laboratories, West Grove, PA, USA; Cat. # 711-166-152; RRID: AB_2313568) and a donkey anti-goat-Alexa488 antibody (1:250, Abcam, Cat. #ab150129; RRID: AB_2687506), at room temperature for 1 h. The sections were then mounted using glass slides in bicarbonate-buffered glycerol at pH 8.6 containing 1 µg/ml DAPI, visualized with a Zeiss Axioplan fluorescence microscope, and photographed with a digital camera.

### RNAseq analysis

100 ng of high-quality total RNA was purified using poly-T magnetic beads. The poly-A mRNA was then fragmented to ~ 200 bp and first- and second-strand cDNA synthesis was done, preserving strand specificity. The resulting double stranded cDNA was converted into adapter-ligated libraries which were amplified, quantitated by qPCR, and sequenced on two 75-cycle NextSeq500 sequencing cartridges to generate the data. Transcript abundances were quantified with Kallisto v0.43.0 ^41^, using the *Mus musculus* GRCm38 cDNA sequences as reference. Kallisto was run with the sequence bias correction and 50 bootstraps. Statistical analysis was done in R v3.3.1^42^. For the parametric analysis, Sleuth^43^ was used for differential gene expression using a linear model with the day and tissue factors as independent explanatory variable, plus a factor interaction term (synergistic effect). A custom filter function was used that keeps any transcript with at least 5 reads in > 47% of samples from any factor-combination. Transcripts passing the Wald test with Benjamini–Hochberg corrected *p* values (a.k.a. false discovery rate, FDR) less than 0.05, and with at least twofold increase or decrease in transcript expression were considered differentially expressed.

### Statistical analysis

For Fig. 1C, data are reported as mean ± SEM. One-way ANOVA was performed followed by Tukey’s multiple comparison test (GraphPad Prism, San Diego, CA, USA). Statistical significance was *p* < 0.05.

## Results

### Verification of microglia-specificity

The RiboTag technology allows isolation of ribosome bound mRNA from microglia as described in the schematic Fig. 1A and in the Methods section. To verify whether the hemagglutinin (HA) expression was limited to microglia in the *Cx3cr1*^*CreER*^*: Rlp22*^*HA*^ mice following tamoxifen injection, we carried out immunofluorescence analysis of the brain tissue of these mice (n = 3). The CX3CR1 + cells in the *Cx3cr1*^*CreER*^ carried GFP and hence microglia were identified by using antibodies to GFP. Immunolabeling of GFP and hemagglutinin revealed that HA expression was exclusively limited to GFP-expressing cells in the subcortical regions examined, including hippocampus, hypothalamus and amygdala. A sample micrograph of the immunohistochemical labeling in amygdala is shown in Fig. 1B.

We further verified the enrichment of cellular markers for astrocytes, microglia, neurons and oligodendrocytes using GFAP, IBA1, MAP2 and MBP respectively in the isolated, microglia-specific IP RNA quantified by qPCR. The enrichment in the microglial IP RNA was compared to the total RNA before IP in 3 animals (Fig. 1C). As expected, we found significant differences in mRNA enrichment (F(3,8) = 8.75, *p* = 0.0066), and Tukey's post-hoc test revealed significant enrichment (*p* < 0.05) of *Iba1* compared to *Gfap, Map2* and *Mbp* with no other significant group differences. The enrichment level of *Iba1* was 135.8 ± 44.34% while those of *Gfap*, *Map2* and *Mbp* were 2.8%, 7.26% and 2.7% respectively. Thus, we were confident that we were isolating microglia-specific RNA.

### The microglial transcriptome is heterogeneous in brain and spinal cord at different disease stages of EAE

Our aim was to characterize the microglial transcriptome in the EAE mice at the pre-onset and symptomatic stages of the disease by next generation sequencing. In our previous work, we showed that emotional behaviour was altered in the EAE mice at pre-onset stage and this was correlated with altered microglial morphology at the electron microscope level in the amygdala. Given that the microglial RNA yield from the amygdala was a limiting factor, we chose to use the subcortical brain regions as a surrogate region. We also harvested the lumbar spinal cord to investigate if there was heterogeneity in the microglial transcriptome between the brain and the spinal cord after EAE induction.

**Figure 2:**
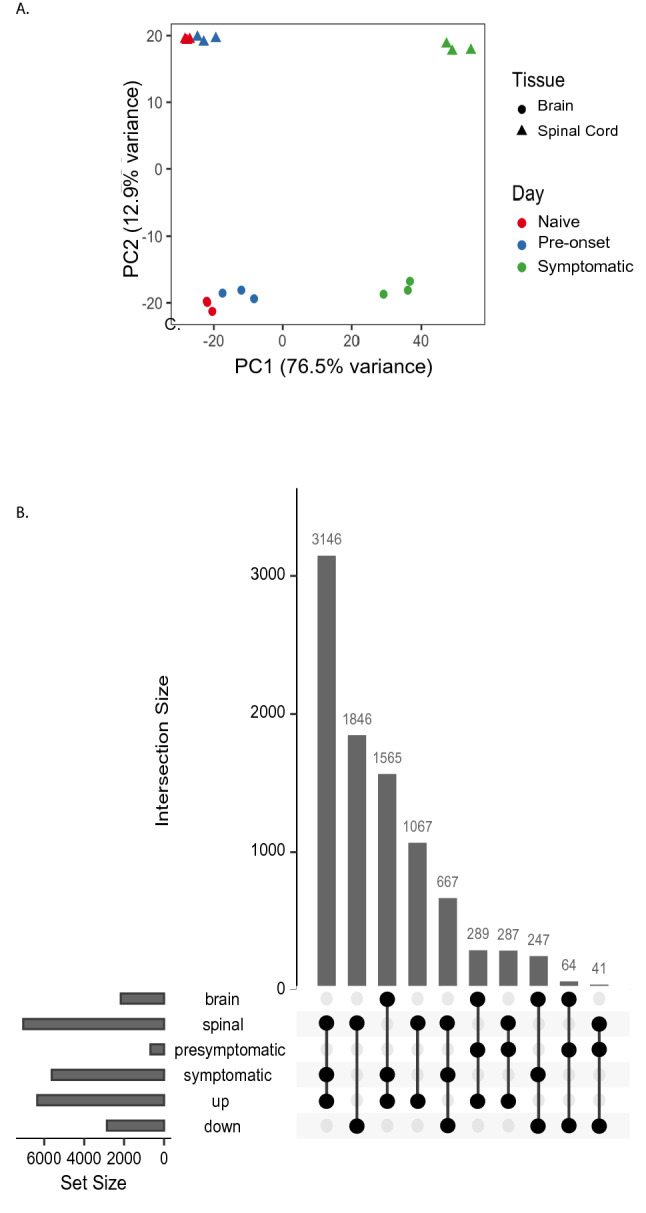
The RNA transcriptome in heterogeneous between the brain and the spinal cord in different disease states. (**A**) Principal component analysis of RNA-seq expression profiles for microglia in the brain and the spinal cord and different disease stages. Separation was observed in the brain and the spinal at different disease stages. The variance in X-axis (PC1) is 76.5% and on Y axis (PC2) is 12.9%. (**B**) UpSeTR diagram showing the number of differentially expressed genes due to each combination of experimental factors using brain in naïve condition as baseline. Three animals per group were used in these experiments^71^.

We used three animals for each condition. We first determined if the microglial transcriptome was heterogeneous based on the tissue and the disease stage. Principal component analysis (PCA), using all mapped genes, showed separation of all the groups and samples clustered in a tissue and disease state dependent manner (Fig. 2A). The distance between the naïve and pre-onset samples was less compared to symptomatic samples, especially for spinal cord. However, the brain and spinal cord samples clustered more distantly, indicating the tissue-based heterogeneity persisted in both the naive and diseased state. The variance in X-axis (PC1) is 76.5% and on Y axis (PC2) is 12.9%.

We compared the expression profile of genes in the brain and the spinal cord at the pre-onset and symptomatic stage to the naïve controls. The genes that were significantly up- or down-regulated (*False Discovery Rate* < 0.05, and two-fold up or down regulation) were chosen for further analysis where they will be referred to as differentially expressed genes (DEGs) from hereon. We first examined if there were any changes at the pre-onset time point in the DEG in the microglia and found that 289 genes were enriched and 64 genes were depleted in the brain, while in the spinal cord 287 genes were enriched and 41 genes were depleted showing that there were roughly comparable number of genes altered in the brain and the spinal cord (Fig. 2B).

In the symptomatic animals, the number of DEGs was several fold higher compared to the pre-onset animals in both brain and the spinal cord. In the symptomatic stage, 3,146 and 1,846 genes were enriched and depleted respectively in the spinal cord, whereas in the brain 1,565 and 247 genes were up- and down-regulated respectively (Fig. 2B).

We ran an analysis to ensure residual macrophages were not influencing differential gene expression, using *Meg3* gene expression level as a proxy for macrophages and found that it did not materially affect the DEGs compared to our existing linear model using the Likelihood Ratio Test (0 gene passing with a False Discovery Rate < 0.05).

### Changes in pre-onset stage of the disease

**Figure 3:**
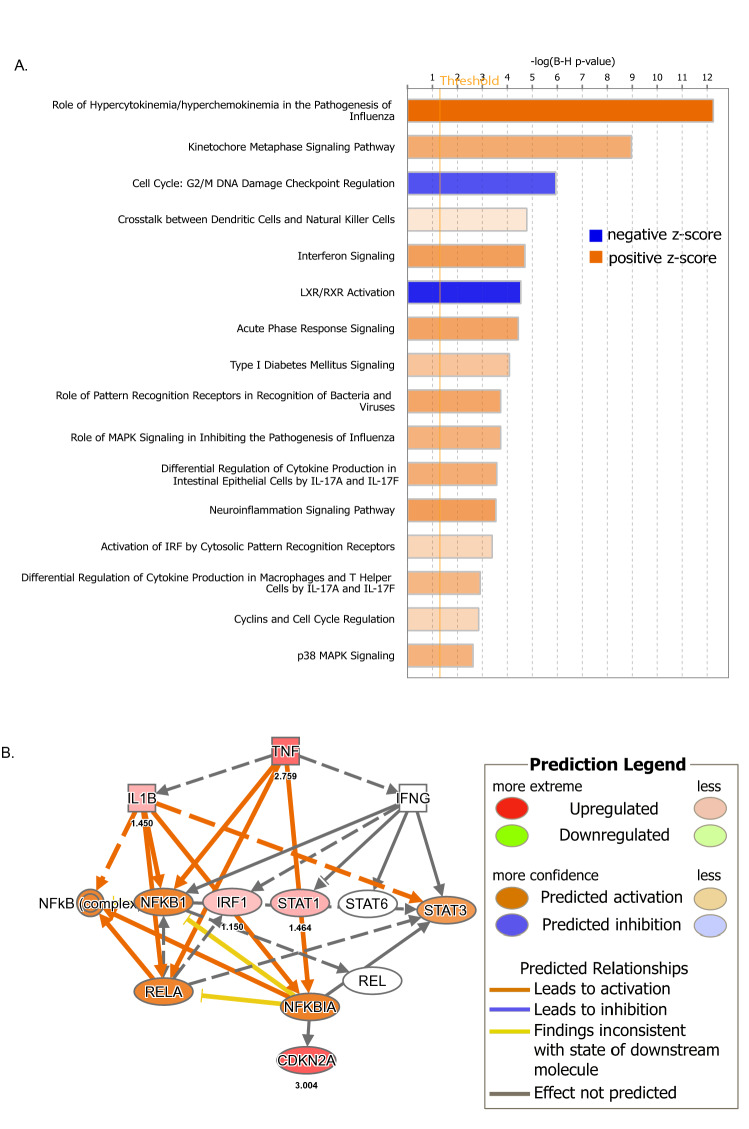
Changes in pre-onset stage of the disease. (**A**) Canonical Pathways from IPA. Bars correspond to the top 16 Canonical Pathways that surpassed the Ingenuity statistical threshold using the Benjamini–Hochberg multiple testing correction of Fisher’s Exact Test in both brain and spinal cord of pre-onset animals. (**B**) Upstream analysis in IPA identified TNF as a master regulator of 169 differentially expressed genes in the experiment and it exerts its effects through 12 intermediary genes. These relationships are depicted in the schematic. The graph and the network were generated through the use of IPA (QIAGEN Inc., https://www.qiagenbioinformatics.com/products/ingenuity-pathway-analysis).

Canonical pathway analysis using Ingenuity Pathway Analysis [IPA] (Qiagen, Redwood City, CA) of the tissues at the pre-onset stage showed that both tissues showed upregulation of 24 pathways and downregulation of three pathways (using a Z-score cutoff of 1.5; Fig. 3A). There was particularly a strong induction of chemokine/cytokine pathways related to the inflammatory response (*p* value 1.54e−15; Z-score 4.123). Seventeen genes in this pathway were upregulated in the process, with the chemokine *Cxcl10* showing the highest expression (Log2 Fold change 2.864) followed by *tnf* (Log2 Fold change 2.759). *Tnf* was the most activated gene for the causal network and IPA showed that this cytokine is a master regulator of 169 of these genes in the chemokine/cytokine signaling pathway (activation Z-score 6.2, *p* value 1.7e−22) and TNF exerts its effect through 12 intermediary factors, including IL1β, IFN-g, NF-kB complex, NF-KB1, IRF-1, STAT1, STAT6, STAT3, RELA, REL, NFkBIA and CDK2A (Fig. 3B, Supplementary Figure 1). There was also a strong down regulation of LXR/RXR activation in both tissues at the pre-onset stage, which is indicative of microglial activation. We also found that pathways related to cell division were altered in both tissues. Particularly, kinetochore metaphase signaling was upregulated (activation Z-score 2.324; *p* value 5.84e−12) and G2/M checkpoint signaling was downregulated (Z-score 1.667; *p* value 1.56e−08), which indicates that there is enhanced cell-proliferation (Fig. 3A).

C3 was upregulated in both brain and spinal cord at the pre-onset stage. Combining the known enrichment of genes involvement in cell motility from IPA knowledge-base with the observed causal network with our gene expression data at pre-onset stage, we posit that C3 has a regulatory effect on cell motility by its effect on 10 genes, namely *Ccl2, Ccl5, Cxcl10, Cxcl2, Il1b, Lcn2, Sell, Selp, Tnf, Zbp1* (IPA consistency score 3.618). Specific to spinal cord at the pre-onset stage, twelve genes (*Adcy1, Agap2, Camk2a, Hivep2, Lcn2, Neurl1, Pak6, Pomc, Psd, Ptgs2, Shank3, Znf365*) related to the formation of cellular protrusions were upregulated (*p* value 4.1e−6, Z-score 2.8).

### Changes in symptomatic stage of the disease

**Figure 4:**
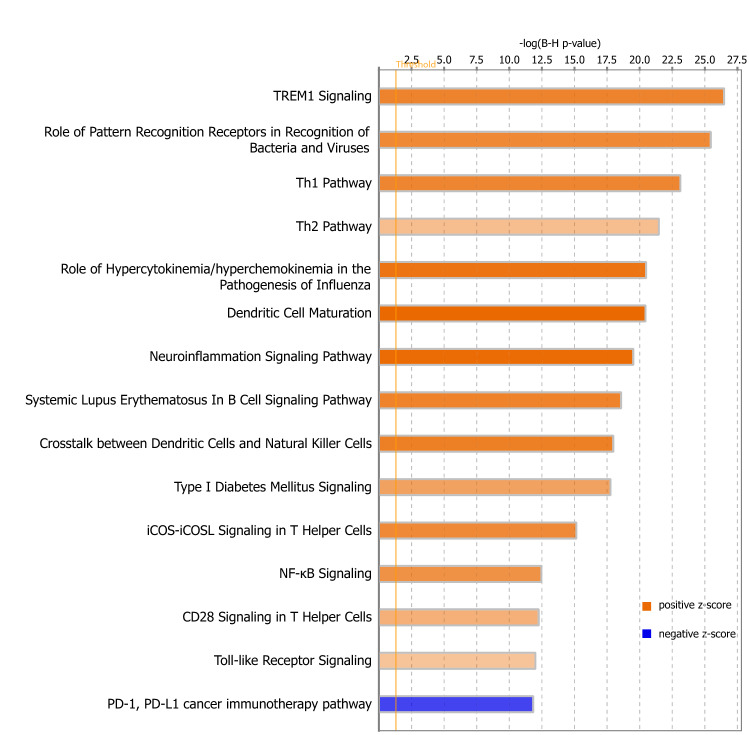
Changes in symptomatic stage of the disease. Canonical Pathways from IPA. Bars correspond to the top 16 Canonical Pathways that surpassed the Ingenuity statistical threshold using the Benjamini–Hochberg multiple testing correction of Fisher’s Exact Test in both brain and spinal cord of symptomatic animals. The graph was generated through the use of IPA (QIAGEN Inc., https://www.qiagenbioinformatics.com/products/ingenuity-pathway-analysis).

In the symptomatic stage of the disease (Fig. 4), both tissues showed upregulation of inflammatory pathways including upregulation of TREM1 signaling (Z-score = 5.55, *p* value = 1.82e−29), pathways linked to pattern recognition receptors (Z-score = 5.303, *p* value = 2.58e−28), hypercytokinemia/hyperchemokinemia (Z-score = 6.245, *p* value = 5.3e−23), neuroinflammatory signaling pathway (Z-score = 6.621, *p* value = 6.25e−22) and Toll-like receptor signaling (Z-score 2.683, *p* value = 2.24e−17). There was also upregulation of T-cell linked pathways, including Th1 pathway (Z-score = 5.466, *p* value = 8.32e−26), Th2 pathway (Z-score = 2.959, *p* value = 4.92e−24) and iCOS-iCOSL signaling in T-helper cells (Z-score = 5.292, *p* value = 2.24e−17). Of note, there was a strong downregulation of PD1 pathways, some of which were related to self-recognition of immune system at the symptomatic stage. This was especially strong in spinal cord. This was on a background of spinal cord generally having higher PD1 activity (Z-score -3.6 for spinal cord and -3.1 for brain).

### Comparison of pre-onset and symptomatic stage of the disease

**Figure 5:**
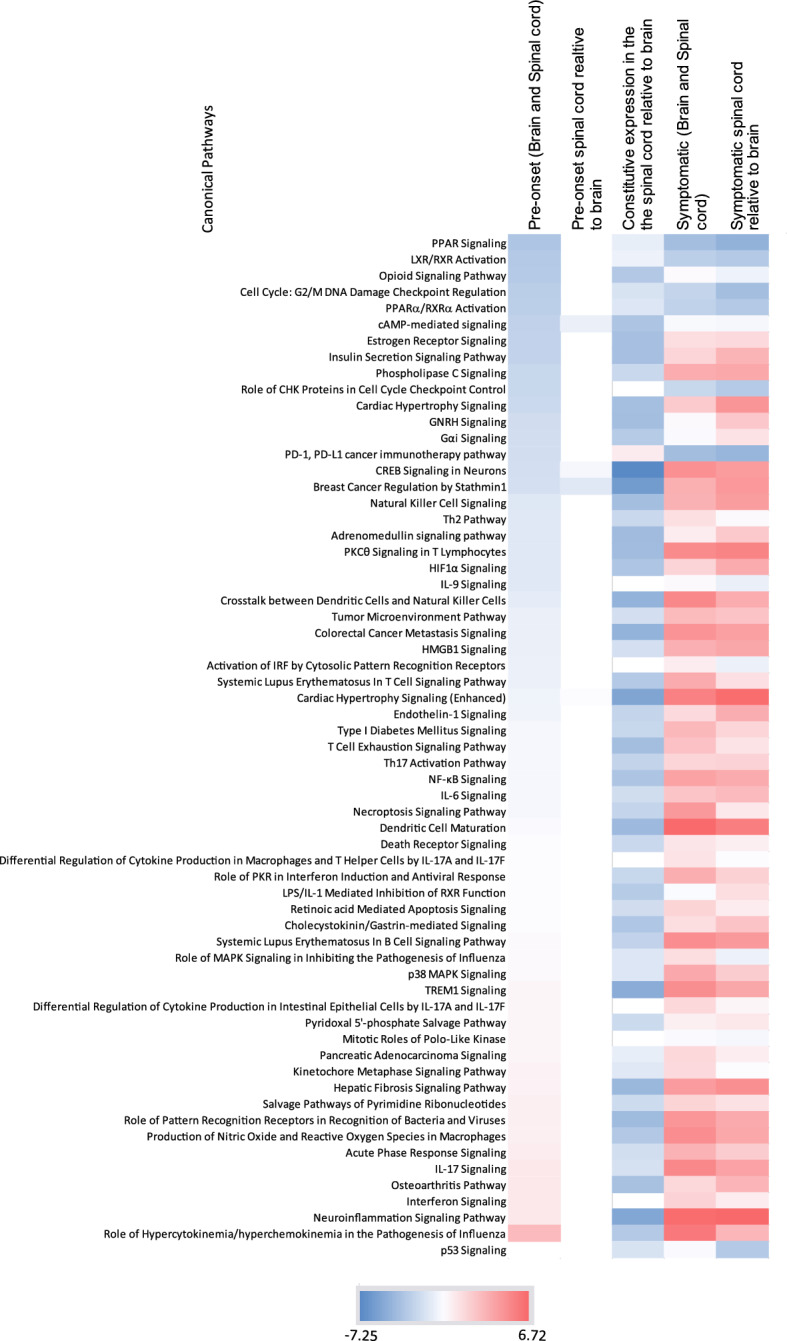
Comparison of the activation of the pathways in the brain and the spinal cord at different stages of the disease. Heatmap of the Z-scores of the canonical pathways in the brain and the spinal cord at the pre-onset and the symptomatic stage of the disease. It is sorted in ascending order of the Z-score for pre-onset brain and spinal cord. p53 pathway has been added to the pre-onset data to highlight the absence of these pathways until symptomatic disease.

Figure 5 shows some of the comparisons of the canonical pathways in the different groups of animals. We observed there is a continuum of many pathways at pre-onset stage into the symptomatic stage. Examples include neuroinflammatory- and cell division-linked pathways. For example, echoing the early signs at the pre-onset stage, we observed a strong downregulation of the G2/metaphase checkpoint regulation pathway in symptomatic brain and spinal cord (Z-score = -3 for spinal cord, and -1.2 for brain). Two main processes—neuroinflammatory pathways and T-cell linked pathways were strongly activated in the symptomatic stage but less so in the pre-onset stage. In terms of neuroinflammation, TREM1 signaling was among the most highly activated. We also found that CREB signaling was much higher in brain than in spinal cord constitutively, but spinal cord showed an increase in the pre-onset stage that became more strongly evident in the symptomatic stage. Additionally, the brain showed a comparable increase in activity at the pre-symptomatic stage which progressed in the symptomatic stage. Interestingly, the p53 pathway showed the very beginnings of upregulation at the pre-onset stage (*Cdkz2a, Birc5, Cdkn1a*), but much stronger induction in both tissue types at the symptomatic stage. A few genes were constitutively less expressed in spinal cord compared to the brain (*Ccdn2, Perp, Tp73, and Rprm*), but additional genes were only downregulated in symptomatic spinal cord (*Pik3c2b, Adgrb1, Plagl1*) which led to opposite p53 pathway activity signals in IPA at symptomatic stage for the two tissues. We also observed changes in IL-17 signaling in EAE, with both tissues strongly affected at the symptomatic stage (Z-score = 5.7) but also an early signal at the pre-onset stage (Z-score = 2.6).

## Discussion

This study has revealed a number interesting findings concerning microglial transcriptional regulation throughout various stages and in various tissues in the EAE mouse model of MS. Principal Component Analysis of the RNAseq data revealed that the brain and the spinal cord samples clustered separately at both stages of EAE. When we compared the gene expression profile between pre-onset and symptomatic EAE mice in the brain and the spinal cord, we observed changes both in the pre-onset and symptomatic stages of EAE that differed somewhat between the brain and spinal cord.

### RiboTag technology

In this study, we isolated microglial transcriptomes from the spinal cord and the brain of EAE animals using RiboTag technology. To the best of our knowledge, this is the first study in which this technology has been exploited to study the transcriptome in the brain and spinal cord microglia in the context of EAE. Haimon et al.^30^ showed that microglial RNA isolated using Ribotag technology was superior to traditional cell-sorting methods in that it avoided incurring artifacts arising from tissue dissociation, cargo contamination and transcripts de-sequestered from ribosomes.

Haimon et al.^30^ showed that the usage of *Cx3cr1*^*CreER*^ mice conferred higher specificity of microglial cell-type expression of the HA-tag compared to *Cx3cr1*^*Cre*^. They found that the HA tag was restricted to microglia when *Cx3cr1*^*CreER*^ mice were used and our immunohistological examination suggested a similar conclusion. A problem when attempting to understand microglial function is that in the symptomatic stage of EAE, macrophages enter the CNS from the periphery. In order to mitigate isolating mRNA from this population of cells, we waited for one month prior to EAE induction so that there would be turnover of macrophages containing the RiboTag^39^. However, we concede that some non-parenchymal brain macrophages may have long half-lives like microglia and hence might still be present with an HA-tag^44^. We addressed this concern by using *Meg3* gene expression level as a proxy for the macrophage and found that its omission or inclusion did not materially affect the DEGs in our existing linear model. Thus, if invading macrophage RNA was in our sample, it was present in insignificant amounts.

The RiboTag technology enabled us to isolate the RNA bound to the ribosome and therefore we focused on the genes that were actively translated and contributing to the cellular translatome. However, one study showed that there is dissociation between the levels of ribosomal bound RNA and the protein level in microglia after an LPS challenge^45^. This study pointed that while there were many genes upregulated at the RNA level, the numbers of upregulated proteins were far less. Thus, we are cautious about our interpretation of the ribosome bound RNA as the translatome. We are unable to isolate only microglial-derived protein in sufficient abundance for quantification of specific proteins, and since the large majority of the RNA we are quantifying from bulk tissue is not microglial-specific, protein analysis by Western blots or ELISAs from whole, multi-cellular tissue would not necessarily apply to microglia; nor would it be able to differentiate between brain/spinal cord produced molecules and those that might arise from the macrophages and other immune cells that cross into the brain through the compromised blood brain barrier in latter stage EAE^46^. Nonetheless, it should be noted that for a number of the microglial predominant DEGs we found to be altered in EAE, the genes or their protein products have previously been shown to be altered, even in whole brain or spinal cord tissue. Relevant examples include products of the complement cascade^47–49^.

It is difficult to compare our data with other studies as no one so far has investigated the differences in microglial gene expression between the different stages of EAE in brain and spinal cord separately. However, it has been shown in microglia isolated from intact brain and spinal cord and subjected to FACS sorting that RNA phenotype changes with disease progression^50^ . We observed many of the same directional changes as well as the specific DEGs identified in this previous study. Below we discuss the changes in different stages of the disease.

### Changes in the pre-onset stage of the disease

Previous data from our lab^19^ showed that there was modest increase only in *Il-1ß* transcripts in the hypothalamus (and not in the hippocampus or amygdala) at the pre-onset stage. However, using the RNAseq strategy, we found that there was increased activation of a number of pathways related to cytokines/chemokines. This indicates that analysis of a specific identifiable cellular population gives a more granular data that may not be apparent in the examination of whole, multi-cellular tissue. Cytokines such as CXCL10 and TNF, that were found to be upregulated in the pathway analysis are secreted by microglia and exert several downstream effects not only on immune regulation, but also, in the case of TNF, on synaptic plasticity, neurogenesis and astrocyte function^51^. For example, it has been shown that TNF changes AMPA receptor current^52^ similar to what we saw in our earlier work^26^. TNF is also an important molecule in cellular immunity, in particular T cell function^53^ and it is no surprise that we found it to be the most activated gene for the causal network. This is in keeping with extensive evidence for secretion of TNF by microglial cells in EAE^54^ and an important role for TNF in the pathology of MS and EAE^55^. CXCL10 is a chemokine that binds to CXCR3 and thus is a chemo-attractant for activated T cells and natural killer cells^56^. There is evidence its upregulation in MS as well as in EAE and its expression in advance of evidence of T cell invasion in the pre-symptomatic stage would be consistent with its role in helping to initiate subsequent T cell invasion^57^.

Complement proteins in the brain are membrane expressed proteins found in microglia that interact with receptors on target cells to initiate phagocytosis. There is now extensive evidence that complement plays an important role in normal brain function^58^, where it has been shown to be causally involved in microglial dependent synaptic pruning^59^. In the pre-onset EAE amygdala, we previously implicated downregulation of C3 in the increased numbers of dendritic spines and augmented excitatory synaptic activity seen at this early stage. This inference was based upon reduced mRNA expression for C3 mRNA, but this did not seem to be localized specifically to the microglia^26^. Likewise, in the current study we saw a generalized upregulation of the complement system in microglia. This is in keeping with an extensive body of evidence reporting upregulation of the complement system in MS and EAE^60^. This was seen in both brain and spinal cord; in the former, it has been implicated in the extensive synaptic pruning and synaptic degeneration seen in these disease states. An upregulation of the complement system at this early stage of EAE has not previously been reported, to the best of our knowledge, but the fact that C3 has a regulatory effect on cell motility, which was a highly upregulated pathway, would be compatible with a role in synaptic pruning. Likewise, the upregulation of numerous genes related to cellular protrusions may point to dynamics of microglial processes that are highly motile both in performing immune surveillance and in engulfing material for phagocytosis.

The pathway analysis revealed that there is upregulation of kinetochore metaphase signaling and downregulation of G2/M damage pathways at the pre-onset stage. These two pathways are related to cell division and the changes in expression of the related genes suggest progression of cell cycle and proliferation of microglia. In resting states, microglia are known to have a low mitotic rate, but their proliferation capacity increases after activation^61^. It has been demonstrated that the rate of microglia proliferation significantly increased in the early stage of EAE before T cell or monocyte infiltration in the CNS^62^. The proliferation of microglia seems to occur specifically early in the pathogenesis and correlates with our finding that genes and pathways regulating cell proliferation were activated during the pre-onset stage of EAE. Although microglia may not play an active role in tissue damage, the proliferation of tissue resident microglia in the early stage of EAE has been shown to be important in subsequent events during the pathogenesis^63, 64^. Thus, it can be speculated that the initial increase in microglial population may be a mechanism to maintain the microglial population in the tissue during the microglial activation.

In an earlier study^32^ where genetically labeled microglia and invading monocytes were flow sorted and RNA expression of a number of inflammation related genes was analyzed at disease onset, there was a general downregulation of a number of metabolic and activation pathways. As noted above, we saw downregulation of pathways linked to cell division (G2/M damage checkpoint regulation) and LXR/RXR signaling, but we did not see a significant alteration of pathways linked to metabolism.

### Changes in the symptomatic stage of the disease

Many more pathways were activated in the symptomatic stage compared to pre-onset. Of note is that using the same twofold cutoff, but applied to specific neuropathology panel encompassing only 770 genes, another study reported differentially expressed genes in whole brain and spinal cord tissue that were markedly lower in number than what we describe here^47^. This may be due to an inability in the previous study to discriminate between cells, for example invading monocytes and microglia that can show divergent expression patterns^32^ and thus effectively cancel each other out, or perhaps may reflect the agnostic nature of the RNAseq in our study that provides a more extensive survey relative to the more targeted neuropathology panel of the earlier study.

TREM1 signaling, which was upregulated in the symptomatic stage, facilitates microglial phagocytosis in some disease conditions^65^ and it is conceivable that TREM1 may also facilitate phagocytosis in EAE models. Interestingly, TREM1 signaling did not cross the statistical threshold in the pre-onset stage. TLR and pattern recognition signaling receptor pathways were also upregulated. We know that these pathways in microglia mediate innate immunity and also link with the adaptive immune response, providing an important mechanism by which microglia are able to sense both pathogen‐ and host‐derived ligands within the CNS^66^. In an acute model of EAE, it has been shown that the activated microglia acquire an immature dendritic cell phenotype and may terminate the immune response^67^. This may be reflected in activation of the pathway linked to dendritic cell maturation. The pathways linked to T-cell signaling may be a reflection of T-cell-microglia interaction that occurs in the EAE. Activated microglia interact with the encephalitogenic T-cells and exacerbate their capacity to induce injury^68^.

### Comparison of the activation of the pathways in the brain and the spinal cord at different stages of the disease

In our study, we could not probe the regional variation within the brain because the ribosome-bound RNA yield from each region using RiboTag technology was very low. We decided to utilize the sub-cortical region of the brain, which includes amygdala, hippocampus, thalamus, basal ganglia and hypothalamus, all being grey matter regions known to be important in behaviour. Concurrently, we analyzed the transcriptome from the spinal cord because demyelination is prominent in the spinal cord of the EAE mouse^10, 69^. There is evidence that T cells enter the CNS through the lumbar region (L_5_) of spinal cord at early stages of EAE^70^. With that knowledge, we expected that we would see increased number of DEGs in the spinal cord compared to the brain in the pre-onset stage. To our surprise, we found that the changes in the brain and the spinal cord were somewhat comparable.

An earlier study compared the pre-onset and symptomatic stages of RNA expression derived from FACS sorted microglial cells and found that oxidative phosphorylation, protein ubiquitination, antigen presentation and mitochondrial dysfunction were all highly enriched in the symptomatic compared to pre-onset group^50^. In our dataset, antigen presentation showed up without any activation pattern (Z-score not applicable) and these differences could be a factor of method of microglial isolation.

## Conclusion

This is the first study to our knowledge that has analyzed the microglial transcriptome in EAE from the brain and the spinal cord using the RiboTag technology. The microglial transcriptome analysis showed up- and down-regulation of many pathways in tissue and disease state dependent manner, thereby opening door for future in-depth analysis on each of these pathways. Of particular importance is the fact that we have identified certain unique alterations in gene expression well before onset of classic motor and sensory symptoms emerge. These findings may be instructive in addressing some of the behavioral issues that are associated with MS even in the absence of active disease symptomology.

## Supplementary Information

Supplementary Information 1.

Supplementary caption.

## Data Availability

All data generated during and/or analyzed during the current study are available upon request to the corresponding author.
