# Supplementary figures and images for: Characterization of microglial transcriptomes in the brain and spinal cord of mice in early and late experimental autoimmune encephalomyelitis using a RiboTag strategy
Source: Sci Rep. 2021 Jul 12;11:14319. doi: 10.1038/s41598-021-93590-1 (PMC8275680; doi:10.1038/s41598-021-93590-1)

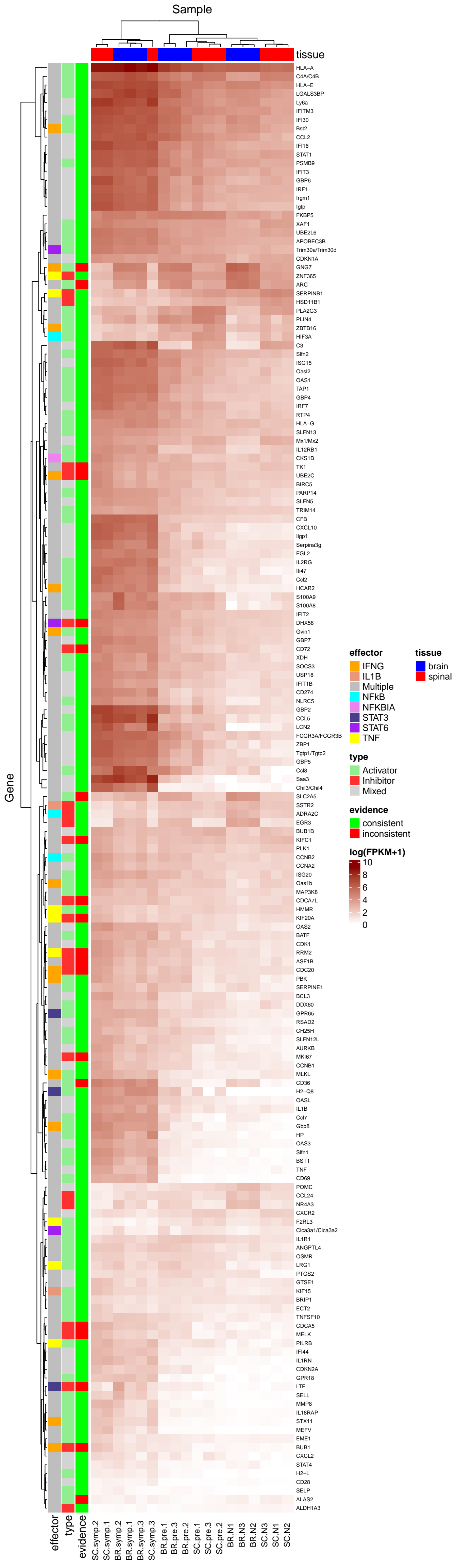

Supplement: Supplementary file 1 — Supplementary Information 1. [file 41598_2021_93590_MOESM1_ESM.pdf]
