# Supplementary material for: Characterization of microglial transcriptomes in the brain and spinal cord of mice in early and late experimental autoimmune encephalomyelitis using a RiboTag strategy
Source: Sci Rep. 2021 Jul 12;11:14319. doi: 10.1038/s41598-021-93590-1 (PMC8275680; doi:10.1038/s41598-021-93590-1)
Supplement: Supplementary file 2 — Supplementary caption. [file 41598_2021_93590_MOESM2_ESM.docx]

**Supplementary Figure 1**. The heat map shows RPKM values for each gene that is regulated by TNF. Each column represents one animal. BR.N = brain naïve; BR.pre = brain pre-onset; BR.symp = brain symptomatic; SC.N = spinal cord naïve; SC.pre = spinal cord pre-onset; SC.symp = spinal cord symptomatic. The row annotations on the left show how these genes are connected to their regulators (effectors in Figure 3b) in the first column. The second column indicates whether the regulatory effect is activating or inhibitory. Finally the third column indicates whether the observed direction of gene expression change is consistent/ inconsistent with the regulatory effects in the literature (via IPA) at the pre-onset stage. Both columns and rows are arranged by default hierarchical clustering using the ComplexHeatmap package in R [72].
